# Sarcolemmal distribution of *I*_Ca_ and *I*_NCX_ and Ca^2+^ autoregulation in mouse ventricular myocytes

**DOI:** 10.1152/ajpheart.00117.2017

**Published:** 2017-05-05

**Authors:** Hanne C. Gadeberg, Cherrie H. T. Kong, Simon M. Bryant, Andrew F. James, Clive H. Orchard

**Affiliations:** School of Physiology, Pharmacology and Neuroscience, University of Bristol, Bristol, United Kingdom

**Keywords:** cardiac myocytes, t-tubules, autoregulation, L-type calcium current, sodium/calcium exchange current

## Abstract

The balance of Ca^2+^ influx and efflux regulates the Ca^2+^ load of cardiac myocytes, a process known as autoregulation. Previous work has shown that Ca^2+^ influx, via L-type Ca^2+^ current (*I*_Ca_), and efflux, via the Na^+^/Ca^2+^ exchanger (NCX), occur predominantly at t-tubules; however, the role of t-tubules in autoregulation is unknown. Therefore, we investigated the sarcolemmal distribution of *I*_Ca_ and NCX current (*I*_NCX_), and autoregulation, in mouse ventricular myocytes using whole cell voltage-clamp and simultaneous Ca^2+^ measurements in intact and detubulated (DT) cells. In contrast to the rat, *I*_NCX_ was located predominantly at the surface membrane, and the hysteresis between I_NCX_ and Ca^2+^ observed in intact myocytes was preserved after detubulation. Immunostaining showed both NCX and ryanodine receptors (RyRs) at the t-tubules and surface membrane, consistent with colocalization of NCX and RyRs at both sites. Unlike *I*_NCX_, *I*_Ca_ was found predominantly in the t-tubules. Recovery of the Ca^2+^ transient amplitude to steady state (autoregulation) after application of 200 µM or 10 mM caffeine was slower in DT cells than in intact cells. However, during application of 200 µM caffeine to increase sarcoplasmic reticulum (SR) Ca^2+^ release, DT and intact cells recovered at the same rate. It appears likely that this asymmetric response to changes in SR Ca^2+^ release is a consequence of the distribution of *I*_Ca_, which is reduced in DT cells and is required to refill the SR after depletion, and NCX, which is little affected by detubulation, remaining available to remove Ca^2+^ when SR Ca^2+^ release is increased.

**NEW & NOTEWORTHY** This study shows that in contrast to the rat, mouse ventricular Na^+^/Ca^2+^ exchange current density is lower in the t-tubules than in the surface sarcolemma and Ca^2+^ current is predominantly located in the t-tubules. As a consequence, the t-tubules play a role in recovery (autoregulation) from reduced, but not increased, sarcoplasmic reticulum Ca^2+^ release.

## Abstract (precis)

This study shows that in contrast to the rat, mouse ventricular Na^+^/Ca^2+^ exchange current density is lower in the t-tubules than in the surface sarcolemma and Ca^2+^ current is predominantly located in the t-tubules. As a consequence, the t-tubules play a role in recovery (autoregulation) from reduced, but not increased, sarcoplasmic reticulum Ca^2+^ release.

t-tubules are invaginations of the surface membrane of cardiac ventricular myocytes that play a central role in excitation-contraction coupling. Contraction is initiated by Ca^2+^ influx [Ca^2+^ current (*I*_Ca_)] through L-type Ca^2+^ channels (LTCCs); this activates ryanodine receptors (RyRs) in the adjacent sarcoplasmic reticulum (SR) membrane to cause Ca^2+^ release from the SR [Ca^2+^-induced Ca^2+^ release (CICR)]. *I*_Ca_, RyRs, and thus CICR occur predominantly at t-tubules (6, 8, 14), which results in a near-synchronous rise in cytosolic Ca^2+^ throughout the cell to levels sufficient to activate the contractile proteins.

For myocytes to relax, Ca^2+^ must be removed from the cytosol. This is achieved by Ca^2+^ reuptake into the SR and Ca^2+^ efflux from the cell. Although SR Ca^2+^ uptake is the main route of Ca^2+^ removal from the cytosol, sarcolemmal Ca^2+^ efflux pathways, the Na^+^/Ca^2+^ exchanger (NCX) and sarcolemmal Ca^2+^ ATPase, also play an important role (1, 25). Evidence largely from rat cardiac myocytes suggests that, like influx, Ca^2+^ efflux also occurs predominantly at the t-tubules (11, 14, 37) where, it has been proposed, NCX has privileged access to Ca^2+^ released from the SR (2, 20, 41).

The balance between sarcolemmal Ca^2+^ influx and efflux determines the Ca^2+^ load of the cell and thus the amplitude of the Ca^2+^ transient and is maintained by a process called “autoregulation,” which involves regulation of both Ca^2+^ influx and efflux by cytoplasmic Ca^2^^+^. For example, sensitizing CICR has only a short-lived effect on the Ca^2+^ transient amplitude (17, 39, 42) because the resulting increase in SR Ca^2+^ release decreases *I*_Ca_ by Ca^2+^-dependent inactivation of *I*_Ca_ and increases Ca^2+^ efflux by stimulating NCX (17, 36). These changes reduce the Ca^2+^ transient amplitude back to baseline levels with an accompanying decrease in SR Ca^2+^ content (17, 39, 42). The role of t-tubules in autoregulation is unknown; however, because *I*_Ca_ and its inactivation by Ca^2+^ as well as NCX current (*I*_NCX_) and its stimulation by Ca^2+^ released from the SR have been reported to occur predominantly at the t-tubules (6, 14, 30), it seems likely that they play an important role in autoregulation. Therefore, this study was designed to determine the sarcolemmal distribution of *I*_Ca_ and *I*_NCX_ and the consequences for the role of the t-tubules in autoregulation in mice.

## MATERIALS AND METHODS

### 

#### Myocyte isolation and detubulation.

Ventricular myocytes were isolated from the hearts of male C57BL/6 mice aged between 11 and 13 wk. All procedures were performed in accordance with United Kingdom legislation and approved by the University of Bristol Ethics Committee. Mice were injected with heparin (500 IU by intraperitoneal injection) and killed by cervical dislocation. The heart was excised and washed in isolation solution supplemented with 0.1 mM CaCl_2_ and 10 U/ml heparin. The heart was then Langendorff perfused with isolation solution for 4 min followed by enzyme solution (isolation solution plus 0.1 mM CaCl_2_, 265 U/ml collagenase, and 0.3 U/ml protease) for ~15 min. The ventricles were then removed and shaken in enzyme solution for 4–6 min before being filtered and centrifuged. Cells were resuspended in isolation solution (pH 7.4) plus 0.1 mM CaCl_2_ and stored for 2–8 h before use on the day of isolation. Detubulation (DT), the physical and functional uncoupling of the t-tubules from the surface membrane, was achieved using formamide-induced osmotic shock, as previously described, by incubating cells with 1.5 M formamide for 2 min before centrifugation and resuspending the cells in Tyrode solution (5). Data from intact and DT myocytes were obtained from separate groups of cells.

#### Chemicals and solutions.

All reagents were obtained from Sigma-Aldrich (Poole, UK) unless otherwise specified. The isolation solution contained (in mM) 130 NaCl, 5.4 KCl, 0.4 NaH_2_PO_4_, 4.2 HEPES, 10 glucose, 1.4 MgCl_2_, 20 taurine, and 10 creatine (pH 7.6 using NaOH). Tyrode solution used for experiments contained (in mM) 133 NaCl, 1 MgSO_4_, 1 CaCl_2_, 1 Na_2_HPO_4_, 10 d-glucose, and 10 HEPES (pH 7.4 using NaOH) plus 5 CsCl to inhibit K^+^ currents. The pipette solution contained (in mM) 100 CsCl, 20 TEACl, 10 NaCl, 0.5 MgCl_2_, 5 MgATP, 10 HEPES, 0.4 GTP-Tris (pH 7.2 using CsOH), and 0.1 pentapotassium salt of the fluorescent Ca^2+^ indicator fluo-4 (Life Technologies, Paisley, UK).

#### Measurement of membrane currents.

Myocytes were placed in a chamber mounted on a Diaphot inverted microscope (Nikon UK, Kingston-upon-Thames, UK). Membrane currents and cell capacitance were recorded using the whole cell patch-clamp technique using an Axopatch 200B patch-clamp amplifier, a Digidata 1440A analog-to-digital converter, and pClamp 10 software (Molecular Devices, Reading, UK), which was also used for data acquisition (at 2 kHz) and analysis. Pipette tip resistances were typically 1.2–2.0 MΩ when filled with pipette solution. All experiments were performed at room temperature.

To monitor Ca^2+^ influx and efflux, and thus Ca^2+^ balance, during a Ca^2+^ transient, holding potential was set to −80 mV; a 500-ms ramp to −40 mV was used to inactivate Na^+^ current followed by step depolarization to 0 mV for 100 ms to activate *I*_Ca_ at a frequency of 1 Hz. *I*_Ca_ was measured as the difference between peak inward current and current at the end of the pulse to 0 mV, and the integral was taken as a measure of Ca^2+^ influx. Inactivation of *I*_Ca_ was quantified by measuring the time to 50% inactivation (*T*_50%_). The current representing Ca^2+^ removed by NCX after the step depolarization (*I*_NCX,tail_) was measured by fitting a single-exponential function to 350 ms of the current trace starting 20 ms after repolarization from 0 to −80 mV and extrapolating back to when the membrane was repolarized. The integral of the exponential was taken as a measure of Ca^2+^ efflux during the Ca^2+^ transient (13, 15). This analysis was performed using MATLAB R2015a (Mathworks, Natick, MA).

To determine the distribution of *I*_NCX_ between the surface and t-tubule membranes, *I*_NCX_ was measured in intact and DT myocytes during the application of 10 mM caffeine to cause spatially and temporally uniform release of SR Ca^2+^ (4); the resulting inward current due to Ca^2+^ extrusion via NCX was recorded at −80 mV, and *I*_NCX_ was taken as the difference between the peak current and the current after caffeine washout.

The distribution of *I*_Ca_, *I*_NCX_, and membrane capacitance (a function of membrane area), and, thus, current density, between the surface and t-tubular membranes was calculated from measurements in intact (whole cell) and DT (surface membrane only) myocytes, as previously described (7, 8). In brief, the currents and capacitance of the surface sarcolemma were calculated from those measured in DT myocytes, corrected for incomplete DT assessed using confocal imaging of Di-8-ANEPPS-stained cells as 12.7%; t-tubular currents and capacitance were calculated from the difference between those in intact cells and those in the surface sarcolemma.

#### Measurement of intracellular Ca^2+^.

Fluo-4 fluorescence was excited at 450–488 nm, and emitted fluorescence was collected at wavelengths <560 nm. Fluorescence was normalized to fluorescence just before application of caffeine (F/F_0_). The rate of decay of Ca^2+^ transients was obtained by fitting single exponential functions to the declining phase of the *I*_Ca_- and caffeine-induced Ca^2+^ transients.

#### Analysis of NCX hysteresis loops.

NCX hysteresis loops were produced by plotting *I*_NCX_ against F/F_0_ during application of 10 mM caffeine to release SR Ca^2+^, as previously described (14, 41). Loops were quantified by calculating the area within the loop and normalizing to the rectangle defined by maximum and minimum *I*_NCX_ and F/F_0_ (41).

#### Immunocytochemistry.

Cells were fixed with 4% paraformaldehyde for 10 min before being permeabilized with 0.1% Triton X-100 and stained with primary antibodies for RyR (MA3-916, Thermo Fisher) or NCX (R3F1, Swant) overnight. Cells were then incubated in Alexa fluor 488-conjugated anti-mouse secondary antibody before being mounted with ProLong Gold. Cells were imaged on an LSM 880 confocal microscope (Zeiss) in Airyscan “super-resolution” mode, with a 1.4 numerical aperture ×63 oil-immersion objective.

Staining at the cell surface and in the center of the cell was determined from a binary cell image obtained using Otsu’s method (27). The perimeter of the cell was outlined manually, and staining within a band that extended 2 µm inside this outline was taken as the cell edge; staining from the image inside this band, excluding the nuclei, was taken as the cell center.

Staining was quantified as follows:

(1) $$\text{Normalized staining density}=\frac{\%\text{bright pixels}}{\%\text{total pixels}}$$

where %bright pixels is the percentage of bright pixels in a given area relative to the total number of bright pixels in the cell and %total pixels is the percentage of pixels in a given area relative to the total number of pixels in the cell.

#### Statistical analysis.

Data are expressed as means ± SE. Errors of derived variables (e.g., t-tubule *I*_Ca_ and *I*_NCX_ densities) and the subsequent statistical analysis were calculated using propagation of errors from the constituent measurements (8). Student’s *t*-tests and two-way ANOVA with the Bonferroni post hoc test were used as appropriate and performed using GraphPad Prism 7 (GraphPad Software, San Diego, CA). The limit of statistical confidence was *P* < 0.05. All statistical tests were performed on the number of cells. Sample sizes (*n* numbers) are given as *c*/*h*, where *c* is the number of cells used from *h* number of hearts.

#### Autoregulation model.

The mathematical model of autoregulation described by Eisner et al. (12) was used to simulate the data obtained in intact and DT myocytes. Baseline values for intact cells were those used by Eisner et al. (12) except that the transsarcolemmal Ca^2+^ efflux fraction (*r*) was decreased from 10% to 8% (the value measured in the current experiments). The relative changes obtained experimentally in DT cells were used to model the data in these cells: *I*_Ca_ was decreased from 10 to 4.1, fractional SR Ca^2+^ release (*f*) was decreased from 0.7 to 0.287, and *r* was maintained at 8%, the value measured in DT cells. Changes in *I*_NCX_ relative to steady state obtained from the experimental data were incorporated for intact and DT myocytes.

## RESULTS

### 

#### I_NCX_ distribution in mouse ventricular cells.

DT significantly decreased cell capacitance from 181 ± 9 (*n* = 28/13) to 128 ± 6 pF (*n* = 24/11, *P* < 0.0001), with no change in cell volume, calculated as previously described previously (3) [54 ± 4 pl (intact) vs. 58 ± 4 pl (DT)]. After correction for incomplete DT, this suggests that 41% of the cell membrane is t-tubular.

**Fig. 1.**
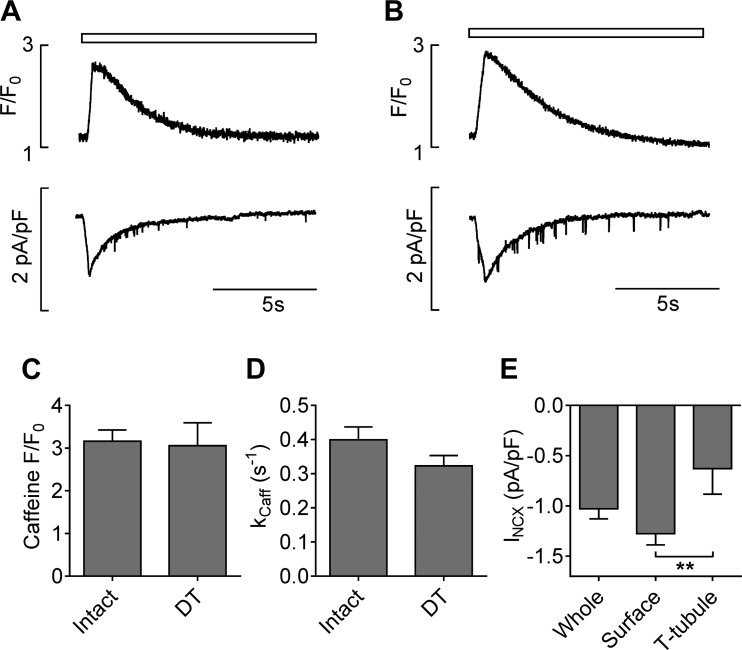
Representative caffeine-induced Ca^2+^ transients (*top*) and corresponding Na^+^/Ca^2+^ exchanger (NCX) current (*I*_NCX_) densities (*bottom*) in intact (*A*) and detubulated (DT; *B*) myocytes. Caffeine application is indicated by the open bars at top. The bar charts show the means ± SE of caffeine-induced Ca^2+^ transient amplitude (*C*), rate of decay (*k*_Caff_; *D*), and *I*_NCX_ density distribution (*E*). Intact: *n* = 14/7; DT: *n* = 10/7. **P < 0.01.

*I*_NCX_ was measured in intact and DT myocytes during application of 10 mM caffeine to determine its distribution. Figure 1 shows representative records of intracellular Ca^2+^, monitored as fluo-4 fluorescence, and the associated *I*_NCX_ in intact (Fig. 1*A*) and DT (Fig. 1*B*) myocytes during application of caffeine. DT had no significant effect on either the amplitude or rate of decay (*k*_Caff_) of the caffeine-induced Ca^2+^ transient (Fig. 1, *C* and *D*), suggesting that SR Ca^2+^ content and sarcolemmal Ca^2+^ efflux are unchanged by DT of mouse cells. Figure 1*E* shows whole cell *I*_NCX_ density and the calculated density of *I*_NCX_ at the surface and t-tubular membranes, showing that the density of *I*_NCX_ in the t-tubules is about half that in the surface membrane (*P* < 0.01). Thus, ~25% of total *I*_NCX_ appears to occur in the t-tubules, consistent with the small, although statistically nonsignificant, decrease in mean *k*_Caff_ on DT (Fig. 1*D*).

**Fig. 2.**
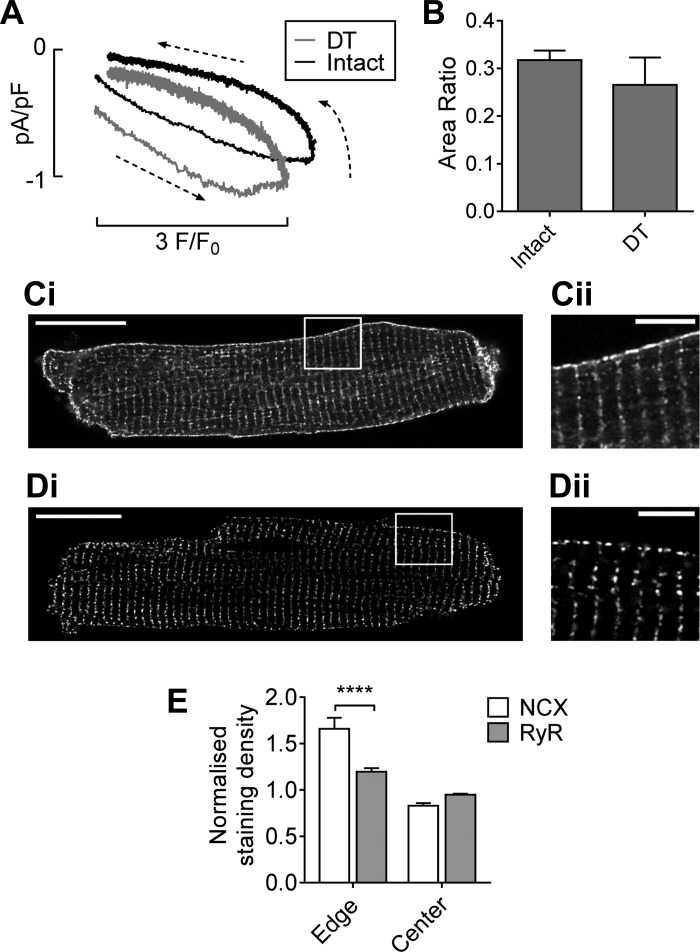
*A*: average hysteresis loops in intact (black; *n* = 14/7) and DT (gray; *n* = 10/7) myocytes. Membrane current is shown relative to that at the end of the application of caffeine in intact myocytes. *B*: mean (±SE) hysteresis area ratios. *C* and *D*: Representative confocal images of NCX (*C*) and ryanodine receptor (RyR; *D*) staining of a whole cell (*i*), with the area indicated shown at higher magnification in *ii*. Scale bars = 20 µm and 5 µm for low- and high-magnification images, respectively. *E*: NCX and RyR staining at the edge and center of the cell calculated according to *Eq. 1*. ****P < 0.0001 with the Bonferroni post hoc test.

Previous work in rat ventricular myocytes has shown that when caffeine is applied to release Ca^2+^ from the SR, *I*_NCX_ is greater for a given cytoplasmic Ca^2+^ concentration as Ca^2+^ increases than during the subsequent decrease. It has been suggested that this hysteresis arises because Ca^2+^ released from the SR has “privileged” access to NCX due to the proximity of NCX to RyRs. Thus, during Ca^2+^ release, NCX is responding to a higher local Ca^2+^ concentration than that reported by a Ca^2+^ indicator in the cytoplasm, while during the falling phase, the local Ca^2+^ concentration surrounding NCX is closer to bulk cytoplasmic Ca^2+^ concentration (41). Hysteresis is lost after DT in rat ventricular myocytes, consistent with the hysteresis arising at the t-tubules as a result of localization of *I*_NCX_ to the t-tubules, close to the site of Ca^2+^ release, in these cells (14, 41). Because the majority of *I*_NCX_ appears to be located at the surface sarcolemma in mouse ventricular myocytes, we investigated whether the hysteresis and its response to DT were different in these cells by plotting *I*_NCX_ against F/F_0_. Figure 2*A* shows that intact cells showed a marked hysteresis that was not abolished by DT: *I*_NCX_ density for a given Ca^2+^ was greater during the rising phase than the declining phase of the caffeine-induced transient in both cell types, with no significant difference in the area ratio of the loop (see Fig. 2*B* and materials and methods). However, the loop was shifted in DT cells, with a greater *I*_NCX_ density for a given Ca^2+^, consistent with a similar Ca^2+^ release but greater *I*_NCX_ density at the cell surface. These data are consistent with the majority of NCX being located at the surface sarcolemma in the mouse and the location of *I*_NCX_ determining the hysteresis, which arises at the site of highest *I*_NCX_ density: the t-tubules in the rat and the surface sarcolemma in the mouse.

#### NCX and RyR distribution.

Immunohistochemistry was used to investigate the distribution of NCX and RyR. Figure 2*C* shows confocal images of a representative mouse myocyte stained for NCX, showing marked striations within the cell and continuous staining at the cell surface, consistent with NCX being present at the t-tubular and surface membranes and thus with the measured distribution of *I*_NCX_. Figure 2*D* shows a representative cell stained for RyRs, which showed marked striations, consistent with t-tubular localization, with less staining at the cell surface, although two or sometimes three distinct areas of RyRs were observed coinciding with the mouth of t-tubules (Fig. 2*D*,*ii*). Staining of NCX and RyRs at the cell edge and cell center, quantified using *Eq. 1*, are shown in Fig. 2*E*; the apparent density of NCX staining was higher than that of RyR at the cell surface (*P* < 0.0001, Bonferroni post hoc test) but similar in the cell center, although both proteins had a greater density at the cell edge than at the cell center (*P* < 0.0001, two-way ANOVA). However, although the images were obtained using Airyscan “super-resolution” to minimize the contribution of out of focus light, there may be more surface membrane than t-tubular membrane in the optical field, since the former is likely to be present in the full depth of the field. This could lead to a higher apparent NCX density at the cell surface, although its effect on measured RyR density is unclear. However, although quantification is difficult, these data show that Ca^2+^ release sites and NCX are present at the cell edge, consistent with the observed distribution of *I*_NCX_ and the hysteresis observed in DT myocytes.

#### I_Ca_ distribution in mouse ventricular cells.

**Fig. 3.**
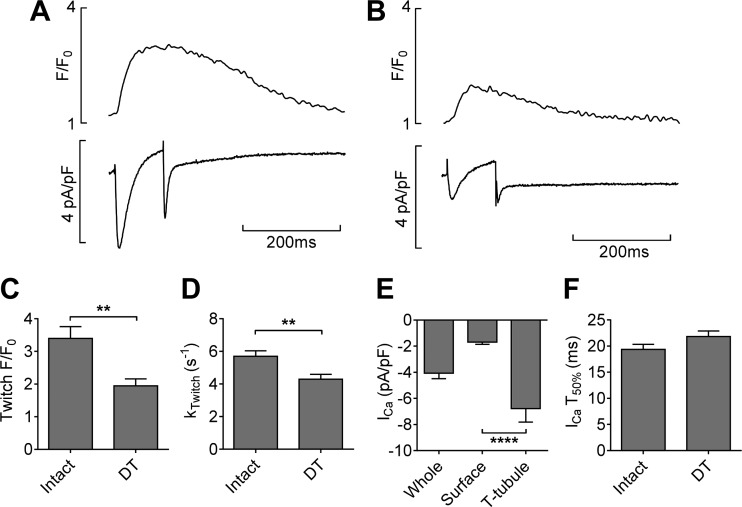
Representative Ca^2+^ current (*I*_Ca_)-stimulated Ca^2+^ transients (*top*) and the corresponding *I*_Ca_ and current representing Ca^2+^ removed by NCX after the step depolarization (*I*_NCX,tail;_ *bottom*) in intact (*A*) and DT (*B*) myocytes. The bar charts show corresponding means (±SE) for Ca^2+^ transient amplitude (*C*), *k*_Twitch_ (*D*), *I*_Ca_ density distribution (*E*), and half-time (*T*_50%_) of *I*_Ca_ inactivation (*F*). Intact: *n* = 14/7; DT: *n* = 10/7. **P < 0.01; ****P < 0.0001.

Since *I*_NCX_ distribution appears to be different in mouse and rat myocytes, we also investigated the distribution of *I*_Ca_ in these cells. Figure 3 shows representative *I*_Ca_ and the elicited Ca^2+^ transients in intact (Fig. 3*A*) and DT (Fig. 3*B*) myocytes. Consistent with previous reports in rat myocytes, the Ca^2+^ transient amplitude (Fig. 3*C*) and rate of decay (Fig. 3*D*) were significantly decreased by DT (5, 6, 21).

*I*_Ca_ in intact and DT cells was used to calculate the distribution of *I*_Ca_ between the surface sarcolemma and t-tubule membranes. Figure 3*E* shows that *I*_Ca_ density was about four times greater in the t-tubule membrane compared with the surface sarcolemma (*P* < 0.0001), as in the rat (6, 8, 14). However, in contrast to the rat, there was no significant change in the rate of inactivation of *I*_Ca_ after DT (Fig. 3*F*), suggesting that inactivation was similar at the surface and t-tubular membranes.

#### Effect of DT on Ca^2+^ autoregulation.

Since the distribution of Ca^2+^-handling proteins determines the role of the t-tubules in Ca^2+^ handling (4, 6, 14, 38), we investigated the effect of detubulation on the recovery of systolic Ca^2+^ transient amplitude and Ca^2+^ flux via *I*_Ca_ and *I*_NCX_ after depletion of SR Ca^2+^ by a high concentration (10 mM) of caffeine, and during and after application of a low concentration (200 µM) of caffeine to sensitize CICR (26, 42), both of which elicit autoregulation.

**Fig. 4.**
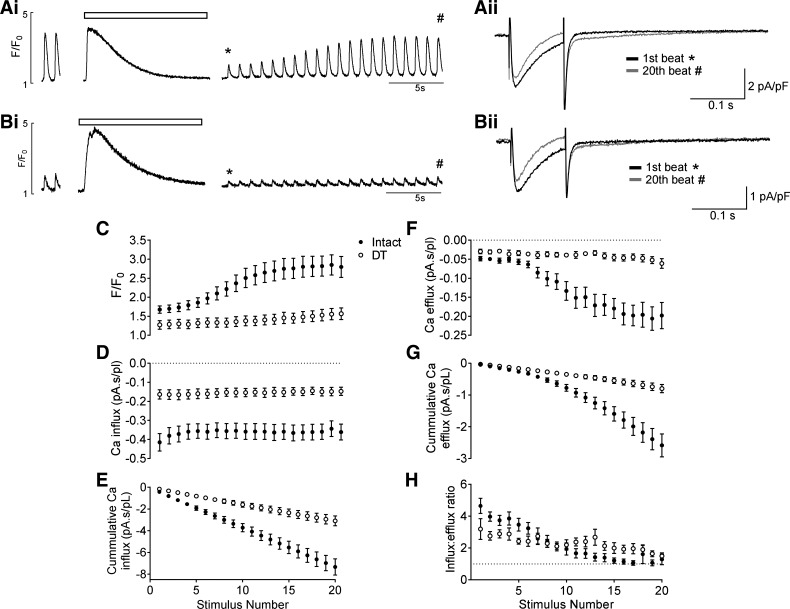
Representative traces showing recovery of the systolic Ca^2+^ transient after application of 10 mM caffeine (shown by the open bars) in intact (*A*,*i*) and DT (*B*,*i*) myocytes. Representative currents for the first (*) and 20th (#) beat are shown in *ii*. *C*: mean Ca^2+^ transient amplitude. DT was significantly different from intact from the 8th stimulus (*P* < 0.05) to the 20th stimulus (*P* < 0.001). *P* values here and subsequently represent the results of a Bonferroni post hoc test. *D*: mean Ca^2+^ influx. DT was significantly different from intact from the 1st stimulus (*P* < 0.0001) to 20th stimulus (*P* < 0.01). *E*: mean cumulative Ca^2+^ influx. DT was significantly different from intact from the 9th stimulus (*P* < 0.05) to 20th stimulus (*P* < 0.0001). *F*: mean Ca^2+^ efflux. DT was significantly different from intact from the 10th stimulus (*P* < 0.05) to 20th stimulus (*P* < 0.0001). *G*: mean cumulative Ca^2+^ efflux. DT was significantly different from intact from the 10th stimulus (*P* < 0.05) to 20th stimulus (*P* < 0.0001). *H*: mean Ca^2+^ influx-to-efflux ratio. *C–H*: all during recovery after caffeine. Intact: *n* = 14/7; DT: *n* = 10/7.

Figure 4 shows that after application of 10 mM caffeine, Ca^2+^ transient amplitude was initially small and gradually recovered to steady state with successive beats in both intact (Fig. *4A*,*i*) and DT (Fig. 4*B*,*i*) myocytes. Recovery was accompanied by a decrease in the amplitude and integral of *I*_Ca_, and an increase in the amplitude and integral of *I*_NCX_ in both intact (Fig. 4*A*,*ii*) and DT (Fig. 4*B*,*ii*) myocytes. However, steady-state Ca^2+^ transient amplitude was significantly smaller in DT cells (*P* < 0.0001), consistent with reduced *I*_Ca_ and loss of t-tubules, and the half-time (*t*_1/2_) to reach steady state was significantly longer (8.7 ± 1.0 s for intact cells vs. 12.6 ± 0.4 s for DT cells, *P* < 0.01); thus, the rate of recovery due to SR refilling was slower in DT myocytes (Fig. 4*C*). Figure 4*D* shows that recovery of Ca^2+^ transient amplitude was accompanied by a small reduction in Ca^2+^ influx via *I*_Ca_ in both intact and DT cells, although Ca^2+^ influx, and thus the rate of Ca^2+^ accumulation, was significantly smaller in DT myocytes (Fig. 4, *D* and *E*). In contrast, Ca^2+^ efflux via *I*_NCX_ gradually increased with continued stimulation in both cell types (Fig. 4*F*), reflecting an increase in SR Ca^2+^ content and release, although Ca^2+^ efflux was significantly smaller in DT cells (Fig. 4, *F* and *G*), which is likely to reflect the decrease in Ca^2+^ transient amplitude due to reduced *I*_Ca_ rather than loss of NCX (above).

The ratio between Ca^2+^ influx and efflux was calculated to compare Ca^2+^ balance in intact and DT cells (Fig. 4*H*). In both cell types, the ratio was initially greater than 1 after caffeine, reflecting net Ca^2+^ influx, and gradually decreased toward 1, which represents the steady-state balance of influx and efflux. However, the initial ratio was slightly lower in DT than intact myocytes. These data suggest that reduced Ca^2+^ influx via *I*_Ca_, and therefore slower Ca^2+^ accumulation, underlies the slower recovery of Ca^2+^ transient amplitude in DT cells and thus that the t-tubules play an important role in autoregulation.

**Fig. 5.**
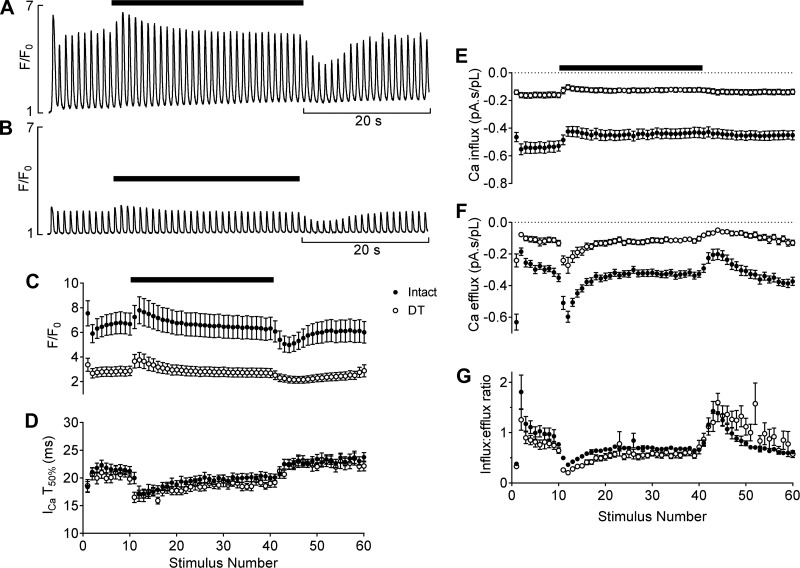
Representative traces showing systolic Ca^2+^ transients before, during, and after application of 200 µM caffeine (shown by solid bar) in intact (*A*) and DT (*B*) myocytes. *C*: mean Ca^2+^ transient amplitude. DT was significantly different from intact on the 1st stimulus (*P* < 0.01), from the 4th to 10th stimulus (*P* < 0.05), 12th to 35th stimulus (*P* < 0.05), 37th (*P* < 0.05), 50th to 53rd stimulus (*P* < 0.05), and 55th and 56th stimulus (*P* < 0.05). *P* values here and subsequently represent the results of a Bonferroni post hoc test. *D*: mean *T*_50%_ of inactivation of *I*_Ca_. *E*: mean Ca^2+^ influx. DT was significantly different from intact for all stimuli (*P* < 0.0001). *F*: mean Ca^2+^ efflux. DT was significantly different from intact for all stimuli (ranging from *P* < 0.01 to *P* < 0.0001) except the 2nd stimulus (not significant). *G*: mean Ca^2+^ influx-to-efflux ratio. DT was significantly different from intact for the 2nd stimulus (*P* < 0.01) and 52nd stimulus (*P* < 0.001). *C–G*: all showing before, during, and after application of 200 µM caffeine (indicated by solid bar). Intact: *n* = 13/6; DT: *n* = 10/4.

To test this idea further, Ca^2+^ autoregulation was investigated during application and washout of 200 µM caffeine to intact (Fig. 5*A*) and DT (Fig. 5*B*) myocytes. Application of caffeine to intact myocytes caused a transient increase in Ca^2+^ transient amplitude, which recovered to steady state, while washout of caffeine caused a transient decrease in Ca^2+^ transient amplitude, which also recovered to steady state, consistent with previous work (e.g., Ref. 39). DT cells showed a similar response, although the Ca^2+^ transient amplitude was smaller; mean data are shown in Fig. 5*C*. Interestingly, the half-time to recover to steady state during application of caffeine was not changed by DT (5.7 ± 0.7 s in intact cells vs. 5.3 ± 0.6 s in DT cells), but the recovery to steady state on washout of caffeine was significantly slowed in DT cells (*t*_1/2_: 3.1 ± 0.3 s in intact cells vs. 5.4 ± 0.4 s in DT cells, *P* < 0.001), similar to the recovery after 10 mM caffeine.

Application of 200 µM caffeine caused similar changes in the rate of inactivation of *I*_Ca_ in intact and DT myocytes (Fig. 5*D*) and decreased Ca^2+^ influx in both cell types, whereas washout caused only a small increase in both cell types (Fig. 5*E*). Ca^2+^ efflux increased on application of 200 µM caffeine, while on washout efflux was reduced (Fig. 5*F*). Although both influx and efflux were significantly (*P* < 0.001) reduced in DT cells, the overall balance was not significantly different (Fig. 5*G*), whether during application 200 µM caffeine, where the balance favored Ca^2+^ efflux, or whether during washout of caffeine, where the balance favored net Ca^2+^ influx.

**Fig. 6.**
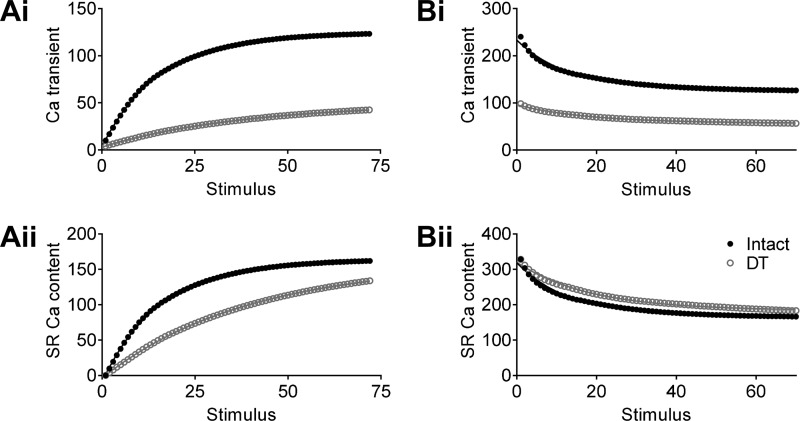
Effect of incorporating the data from intact and DT myocytes into the model of Eisner et al., showing the effects of detubulation on autoregulation. Shown is the recovery of the Ca^2+^ transient (*i*) and sarcoplasmic reticulum (SR) Ca^2+^ content (*ii*) after depletion of SR Ca^2+^ (*A*) and during sensitization of Ca^2+^-induced Ca^2+^ release (*B*).

Taken together, these data suggest that t-tubules play an important role in recovery from a decrease in SR Ca^2+^ load, since DT cells recover more slowly, but not in recovery from an increased SR Ca^2+^ release, since intact and DT cells recover at similar rates. To test this idea further, we incorporated the data from intact and DT myocytes into the model of Eisner et al. (12), as described in materials and methods. The results of these simulations are shown in Fig. 6, which shows that after SR Ca^2+^ depletion (Fig. 6*A*), DT cells recovered much more slowly than intact cells (*t*_1/2_: 10.7 stimuli in intact cells vs. 26.8 stimuli in DT cells), as observed experimentally (cf. Fig. 4*C*). In contrast, the rate of recovery during sensitization of CICR (Fig. 6*B*) was similar in intact and DT myocytes (*t*_1/2_: 8.8 stimuli in intact cells vs. 12.8 stimuli in DT cells), consistent with the data from the experiments (cf. Fig. 5*C*).

## DISCUSSION

The present study was designed to investigate the role of the t-tubules in Ca^2+^ autoregulation in mouse ventricular myocytes. The data show that the majority of *I*_Ca_ occurs in the t-tubules, whereas, in contrast to rat myocytes, *I*_NCX_ occurs mainly at the surface sarcolemma and the hysteresis between Ca^2+^ and *I*_NCX_ persists after DT. Although autoregulation to steady state occurred after DT, the time course of recovery was slower during recovery from decreased SR Ca^2+^ release but, interestingly, not from increased SR Ca^2+^ release. This suggests that t-tubules play a role in recovery from decreased, but not increased, SR Ca^2+^ release, which may reflect the localization of *I*_Ca_ and *I*_NCX_.

### 

#### Distribution of I_Ca_ and I_NCX_ in the mouse sarcolemma.

Previous work has shown that *I*_Ca_ and *I*_NCX_ occur predominantly in the t-tubules in rat ventricular myocytes (6, 8, 11, 14, 21, 37, 44). The present study demonstrated a slightly higher t-tubular membrane fraction (41%) than that previously reported for rat myocytes (6, 8, 30), consistent with the higher t-tubule density reported previously for the mouse (18, 29); it also showed that *I*_Ca_ is located predominantly in the t-tubules of mouse myocytes, consistent with a previous report using DT in mouse myocytes (19). The presence of *I*_Ca_ at the t-tubules in both species, in close proximity to the majority of RyRs, allows the tight coupling between Ca^2+^ influx and SR Ca^2+^ release that underlies CICR, while the higher t-tubule density in the mouse may reflect its high heart rate and the consequent need for rapid activation.

However, the present work also demonstrated that, in contrast to rat ventricular myocytes, *I*_NCX_ is located predominantly at the cell surface in mouse myocytes. Although this might explain some of the discrepancies in the literature regarding the location of NCX determined using immunological techniques (35, 37), it is unclear why the distribution of *I*_NCX_ should be different in the two species, which have similar Ca^2+^-handling properties, with similar action potential configurations, fractions of SR and trans-sarcolemmal Ca^2+^ flux (1, 10, 22, 25), and kinetics of contraction and relaxation (23), although computer modeling suggests that NCX distribution has relatively little effect on whole cell Ca^2+^ handling (31). One possibility is that location of NCX at the cell surface is energetically favorable, which might be important at the higher heart rates in the mouse, because it will avoid the futile Ca^2+^ cycling that results from NCX being in close proximity to the main site of SR Ca^2+^ release, by decreasing the amount of released Ca^2+^ that is immediately removed via NCX, enabling more of the released Ca^2+^ to activate the contractile proteins. The small t-tubular *I*_NCX_ is, however, associated with a small (nonsignificant) decrease in the rate of decay of the caffeine-induced Ca^2+^ transient after DT in the mouse, in contrast to the marked decrease observed in the rat (14).

This species difference in the sarcolemmal distribution of Ca^2+^-handling protein function raises questions about the distribution in other species. Although, as far as we are aware, corresponding data are not available for large mammals, previous work in cultured guinea pig myocytes (28) showed no significant change in *I*_NCX_ density with time in culture, whereas cell capacitance decreased (correlating with loss of t-tubules), suggesting a similar *I*_NCX_ density in the surface sarcolemma and t-tubules of guinea pig myocytes (28). Nevertheless, the maintained *I*_NCX_ density might have been due to other mechanisms upregulating *I*_NCX_ in culture.

It has been suggested that the hysteresis between *I*_NCX_ and bulk cytoplasmic Ca^2+^ is due to the proximity of NCX to the site of Ca^2+^ release, so that the exchanger responds to a higher Ca^2+^ concentration than monitored in the bulk cytosol during Ca^2+^ release. The hysteresis is abolished after DT of rat myocytes (14), suggesting that this occurs at the t-tubules, where the majority of NCX is located close to the site of SR Ca^2+^ release, and that surface sarcolemmal *I*_NCX_ responds mainly to changes in global Ca^2+^ concentration. The present observation that the hysteresis is present in both intact and DT mouse myocytes is consistent with the observed distribution of *I*_NCX_ in these cells and suggests that *I*_NCX_ at the surface sarcolemma is located close to sites of Ca^2+^ release and responding to local changes of Ca^2+^ concentration during Ca^2+^ release (and to changes in global Ca^2+^ concentration during the descending phase). The staining data support this idea: RyRs were observed not only in striations in the cell interior, consistent with localization at the t-tubules, but also at the surface membrane in clusters at the mouth of t-tubules, close to NCX, which, in agreement with the *I*_NCX_ measurements, was also observed at both the t-tubules and cell surface. These data suggest that RyRs, and thus Ca^2+^ release, occur at the t-tubules and cell surface and thus that it is the distribution of *I*_NCX_ that determines the site of the hysteresis in these cells. These data also suggest that the site of origin of arrhythmogenic delayed afterdepolarizations, which are caused by activation of *I*_NCX_ by SR Ca^2+^ release, will depend on the location of *I*_NCX_ and Ca^2+^ release and may thus be different in different species.

#### Effects of DT on autoregulatory mechanisms.

The present data show that, in agreement with previous studies in other species (17, 39), recovery of Ca^2+^ transient amplitude following altered SR Ca^2+^ release is associated with reciprocal changes in Ca^2+^ influx via *I*_Ca_ and Ca^2+^ efflux via NCX.

Changes in Ca^2+^ transient amplitude result in changes in Ca^2+^ influx through Ca^2+^-dependent inactivation of *I*_Ca_. In rabbit cells, Ca^2+^ influx can increase by 50–60% in the absence of SR Ca^2+^ release (16, 33); in the rat, Ca^2+^ influx can double after SR Ca^2+^ depletion (40). Although Ca^2+^-dependent inactivation also occurs in mouse myocytes, as seen by the faster inactivation during application of 200 µM caffeine and slower inactivation on wash off in the present study, only small differences in the rate of inactivation between intact and DT myocytes were observed. This suggests, in contrast to rat myocytes in which Ca^2+^-dependent inactivation occurs predominantly at the t-tubules (6), that inactivation is similar at the surface and t-tubular membranes. The reason for this species difference is unknown but may reflect differences in local Ca^2+^ and/or local LTCC regulation at the two sites. The lack of change in the rate of inactivation of *I*_Ca_ after DT, despite the smaller Ca^2+^ transient, could be explained by the absence of basal Ca^2+^-dependent inactivation of *I*_Ca_ or by rapid inactivation, which is manifested as a change in peak *I*_Ca_ rather than its duration. However, studies using intracellular Ca^2+^ chelators or barium as the charge carrier for *I*_Ca_ to inhibit Ca^2+^-induced inactivation have suggested that there is substantial basal *I*_Ca_ inactivation, which mainly affects the rate of inactivation rather than peak *I*_Ca_ (32, 34, 43), making these explanations unlikely. Thus, the most likely cause for *I*_Ca_ inactivation being unaffected by DT appears to be that local Ca^2+^ release, and thus inactivation, at the cell surface is similar to that at the t-tubules. In this case, the smaller, slower Ca^2+^ transient in DT myocytes is due to loss of the quantitatively more important t-tubular CICR and loss of synchronization of Ca^2+^ release. A similar local Ca^2+^ release at the cell surface and t-tubules can explain why the hysteresis occurs in DT myocytes given the observed distribution of *I*_NCX_. Thus, it appears that, in contrast to the rat, both *I*_NCX_ density and Ca^2+^-dependent inactivation of *I*_Ca_ are similar at the t-tubular and surface membranes and that the decrease in Ca^2+^ influx after DT is predominantly due to loss of t-tubular *I*_Ca_ rather than an altered rate of inactivation.

In contrast to *I*_Ca_, regulation of *I*_NCX_ appears similar to that previously reported, with an increase in *I*_NCX_ associated with an increase in Ca^2+^ transient amplitude in intact and DT myocytes. DT resulted in changes in *k*_Caff_ and *I*_NCX_ consistent with loss of the t-tubular fraction of *I*_NCX_ (25%), while the hysteresis between *I*_NCX_ and cytosolic Ca^2+^ showed a larger current for a given Ca^2+^ in DT myocytes, with no loss of hysteresis, consistent with the greater density of *I*_NCX_ in the surface membrane and similar Ca^2+^ release at the surface and t-tubular membranes. Thus, the observed changes are consistent with the observed distribution of *I*_NCX_, with no evidence for altered regulation.

#### Effects of DT on autoregulation.

As in previous studies (4, 14), DT had no effect on SR Ca^2+^ content, as assessed by releasing SR Ca^2+^ using 10 mM caffeine, but did result in a smaller and slower voltage-stimulated Ca^2+^ transient, due to decreased *I*_Ca_ and thus CICR and loss of synchronization of SR Ca^2+^ release. The smaller Ca^2+^ transient, in turn, decreased *I*_NCX_, even though it was present at the surface sarcolemma.

Although DT had no effect on the rate of recovery of Ca^2+^ transient amplitude to steady state when SR Ca^2+^ release was increased using low-dose caffeine, recovery was slower from a decreased SR Ca^2+^ content after caffeine-induced SR Ca^2+^ depletion or on washout of 200 µM caffeine. This asymmetric response to changes in SR Ca^2+^ release in DT cells was not due to the nonlinear response of fluo-4 to Ca^2+^ because it was still present after the conversion of fluorescence to Ca^2+^ (not shown), an idea supported by the model. It may, however, be explained by the differential distribution of *I*_Ca_ and *I*_NCX_. In this case, when an increase in SR Ca^2+^ release occurs, Ca^2+^ efflux via NCX is the main mechanism to decrease Ca^2+^ transient amplitude and return the cell to steady state; thus, in DT mouse cells, in which the majority of *I*_NCX_ occurs at the surface, recovery is similar in intact and DT myocytes and is not affected by the loss of *I*_Ca_. However, when SR Ca^2+^ release is decreased, Ca^2+^ influx via *I*_Ca_ is necessary to refill the SR and increase Ca^2+^ transient amplitude to steady state and is thus essential for recovery. Since steady-state SR Ca^2+^ content is similar in intact and DT myocytes but Ca^2+^ influx via *I*_Ca_ is smaller in DT cells, more stimuli are required to refill the SR to steady state, so that recovery is slower in DT than in intact cells, even though the majority of NCX is present and quantitatively shows greater changes over time than *I*_Ca_. Slowing of recovery from caffeine has also been reported after DT of rat myocytes (4), consistent with a key role for *I*_Ca_, which, like *I*_NCX_, occurs predominantly in the t-tubules in this species.

Although t-tubules uncoupled from the surface membrane by DT may reseal within the cell and, in principle, sequester and release Ca^2+^ (5, 24), it seems unlikely that they can explain the current data, since they are electrically uncoupled from the surface membrane. Therefore, Ca^2+^ channels in such resealed tubules will not undergo activation (and thus inactivation), an idea supported by the lack of Ca^2+^ release in the center of DT myocytes (5). Because they do not release Ca^2+^ and have a small volume, they are also unlikely to take up Ca, and previous work inhibiting surface NCX in DT rat myocytes during application of caffeine showed that only ~2% of Ca^2+^ removal occurs into non-SR sinks within the cell (9), which includes mitochondria, and this is likely to be even less in mouse myocytes, which have a smaller fraction of NCX within the t-tubules. Thus, resealed t-tubules are unlikely to play an important role in normal Ca^2+^ cycling or autoregulation, an idea supported by the effects of DT on the Ca^2+^ transient and autoregulation.

Thus, it appears that the t-tubules play an important role in autoregulation in mouse myocytes during recovery from decreased SR Ca^2+^ release, because of the high t-tubular *I*_Ca_ density, but play little role in the recovery from increased Ca^2+^ release. These data also suggest that the response of a particular species to such changes will depend on the distribution of *I*_Ca_ and *I*_NCX_ between the t-tubules and surface sarcolemma. Although the distribution of Ca^2+^ fluxes in human myocytes is unknown, the current work shows that the distribution of *I*_Ca_ and *I*_NCX_ determines cell function and enhances our understanding of how this occurs. Thus, when human data become available, it should be possible to better predict the consequent changes in cell function in both physiological conditions and after t-tubule disruption and changes in the distribution of *I*_Ca_ and *I*_NCX_, which have been reported in heart failure (14) and which may also affect autoregulation.

## GRANTS

This work was supported by British Heart Foundation Grants PG/14/65/31055 and RG/12/10/29802.

## DISCLOSURES

No conflicts of interest, financial or otherwise, are declared by the authors.

## AUTHOR CONTRIBUTIONS

H.C.G. performed experiments; H.C.G. and C.H.K. analyzed data; H.C.G., S.M.B., A.F.J., and C.H.O. interpreted results of experiments; H.C.G. prepared figures; H.C.G. and C.H.O. drafted manuscript; H.C.G., C.H.K., S.M.B., A.F.J., and C.H.O. edited and revised manuscript; A.F.J. and C.H.O. conceived and designed researc h; A.F.J. and C.H.O. approved final version of manuscript.
